# Gastric Submucosal Tumor in Patient Infected with *Dioctophyme renale* Roundworm, South Korea, 2024

**DOI:** 10.3201/eid3109.241944

**Published:** 2025-09

**Authors:** Dong-Min Kim, Youngdae Kim, Jung In Lee, Jun-Won Seo, Da Young Kim, Na Ra Yun, Beomgi Lee, You Mi Lee, Choon-Mee Kim, Sung-Chul Lim

**Affiliations:** Author affiliation: Chosun University College of Medicine, Gwangju, South Korea

**Keywords:** parasites, zoonoses, Dioctophyme renale, roundworm, giant kidney worm infection, gastrointestinal stromal tumors, anisakiasis, genetic association studies, South Korea

## Abstract

We describe a case of a gastric submucosal tumor in a patient in South Korea infected with *Dioctophyme renale* roundworm*.* The patient had a history of consuming raw freshwater fish. Molecular and morphologic analyses confirmed *D. renale* Infection. Genetic testing should be used to diagnose rare parasitic infections with unusual clinical manifestations.

The giant kidney worm (*Dioctophyme renale*) is the largest parasitic roundworm and infects the kidneys of carnivorous mammals such as mink (family Mustelidae) (*1*). *D. renale* roundworms are found worldwide except in Africa. Female worms reach up to 103 cm long, and male worms reach up to 45 cm long (*2*). At least 49 mammal species, including humans, have been identified as definitive hosts of *D. renale* roundworms (*2*). Humans typically acquire *D. renale* infection from consuming raw or undercooked freshwater fish or frogs.

Mustelid mammals are the most common definitive hosts of *D. renale* roundworms, although infection may occur in other carnivorous mammals, including canids, raccoons, and otters. Adult male and female worms reside and sexually reproduce in the kidneys of the host. The eggs are excreted in the urine and, once reaching a freshwater environment, develop into first-stage larvae within ≈1 month. Those larvae are ingested by oligochaete worms (phylum Annelida), the intermediate hosts, in which they develop to infective third-stage (L3) larvae. Paratenic hosts, such as fish, frogs, and toads, become infected by consuming the oligochaetes, which harbor encysted L3 larvae. When a carnivorous mammal consumes an infected paratenic host, L3 larvae migrate from the gastric wall to the liver and preferentially infect the right kidney, where they mature into adult worms and complete their life cycle (*3*). We report *D. renale* roundworm infection in a woman in South Korea who had a gastric submucosal tumor.

## The Study

A 53-year-old woman sought care for epigastric pain that worsened on an empty stomach and improved with eating. She had no notable medical history. Two weeks before admission, she had consumed raw smelt (*Hypomesus olidus*) and far-eastern catfish (*Silurus asotus*) stews. A gastroendoscopy 4 days before admission revealed a 2-cm mass in the stomach, prompting referral to the Department of Gastroenterology at Chosun University Hospital (Gwangju, South Korea).

**Figure 1:**
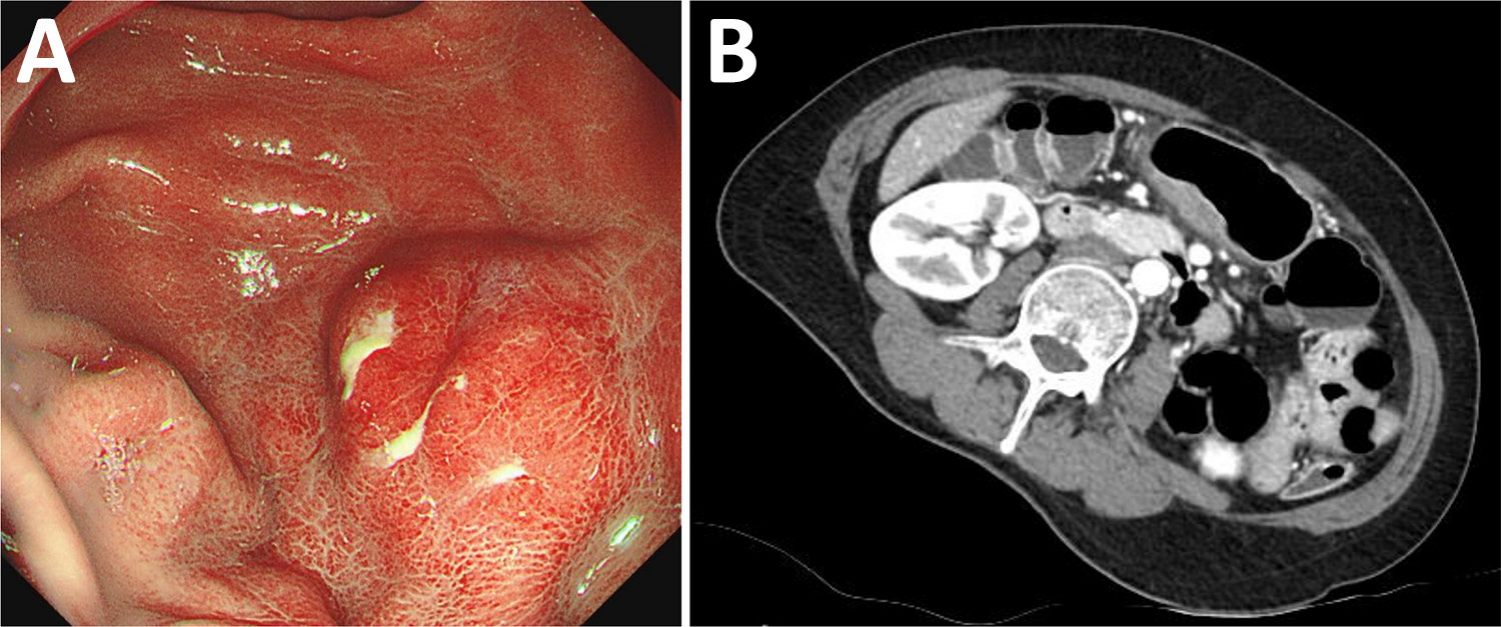
Gastric submucosal tumor in patient infected with *Dioctophyme renale* roundworm, South Korea, 2024. A) Gastroscopy showed a protruding mass-like lesion measuring 3 × 2 cm on the posterior gastric wall. B) Abdominal computed tomography showed enhanced wall thickening and a central low-attenuation area at the greater curvature of the gastric antrum.

Examination showed a 3 × 2 cm protruding mass in the stomach, accompanied by cheese-like exudates and edematous changes in the posterior wall of the proximal gastric antrum (Figure 1). Endoscopic ultrasound showed diffuse thickening (3.2 × 1.2 cm) and disruption of the submucosal layer (Appendix Figure 1). A biopsy showed a 1.8-cm roundworm, initially suspected to be a transmural malignancy (Appendix Figure 2). We then referred the patient to an infectious disease clinic. The worm was successfully removed during endoscopic biopsy, and follow-up revealed no additional symptoms. 

**Figure 2:**
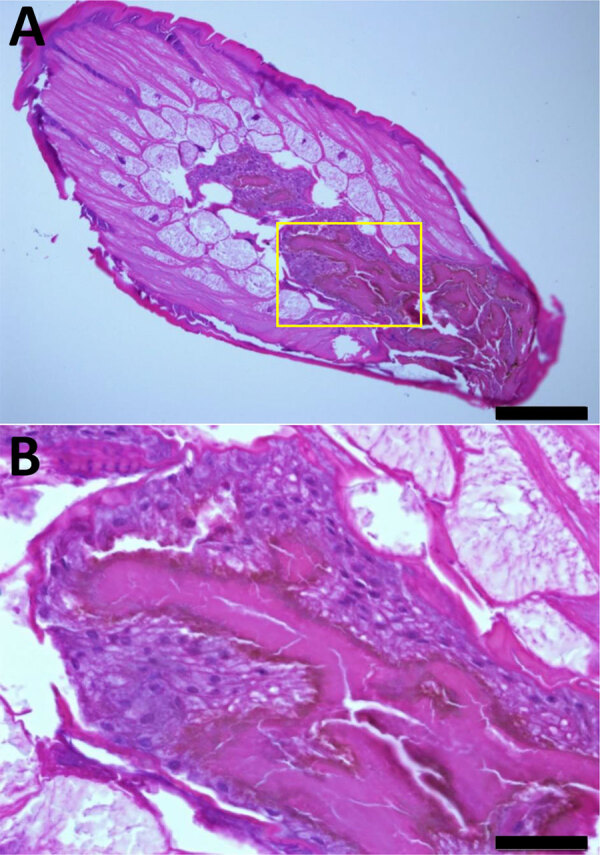
*Dioctophym renale* larva stained with hematoxylin and eosin in a study of gastric submucosal tumor in patient infected with *D. renale* roundworm, South Korea, 2024. A) Cross-section displays long subcuticular polymyarian musculature and characteristic intestines. Spines were not observed on the surface of the body. The intestinal epithelium consists of multilayered cuboidal cells with relatively large nuclei. Numerous dark brown granules (hematoxylin and eosin stain) are visible along the luminal border and are covered with microvilli. Scale bar indicates 200 μm. B) Higher magnification of the specimen shown in panel A (yellow box), providing a closer view of the characteristic intestine. These images suggested the presence of *D. renale* L3 larva. Scale bar indicates 2050 μm.

Laboratory results showed a leukocyte count of 5,060 cells/mm^3^ (reference range 4,000–10,800 cells/mm^3^), eosinophil count of 230 cells/mm^3^ (reference range 0–500 cells/mm^3^), hemoglobin level of 13.2 g/dL (reference range 13–17 g/dL), and total IgE level of 52.5 IU/mL (reference range 0–358 IU/mL). ELISA revealed IgG titers of 0.328 for *Paragonimus westermani* (lung fluke) and 0.417 for *Clonorchis sinensis* (Chinese liver fluke). Results of a routine fecal examination was negative for parasites. The cross-section of the dark red roundworm showed an absence of surface spines and the presence of long polymyarian musculature and multilayered cuboidal intestinal cells with large nuclei (Figure 2).

We performed PCR by using primers designed against reference sequences of the roundworm (Appendix Table). The initial differential diagnoses included *Anisakis* spp., *Gnathostoma* spp., and *D. renale* roundworms on the basis of the patient’s history and clinical manifestations. We ruled out *Anisakis* spp. infection on the basis of negative PCR results targeting the cyclooxygenase-2 gene (*4*). Next, we considered *Gnathostoma* spp. Infection but ruled it out because small subunit rRNA gene sequencing showed 100% (974/974 bp) homology with *D. renale* (GenBank accession nos. OR501903, OQ933019, and OQ918640), whereas sequence identity with *G. spinigerum* (GenBank accession no. MT879607) was only 79.05% (717/907 bp). Additional nested PCR confirmed 100% (561/561 bp) homology with *D. renale*, further excluding *Gnathostoma* (83.83% identity [337/402 bp]) with *G. spinigerum* (GenBank accession no. MT879607). We deposited the confirmed sequences in GenBank (accession nos. PP981196 and PV168478). 

**Figure 3:**
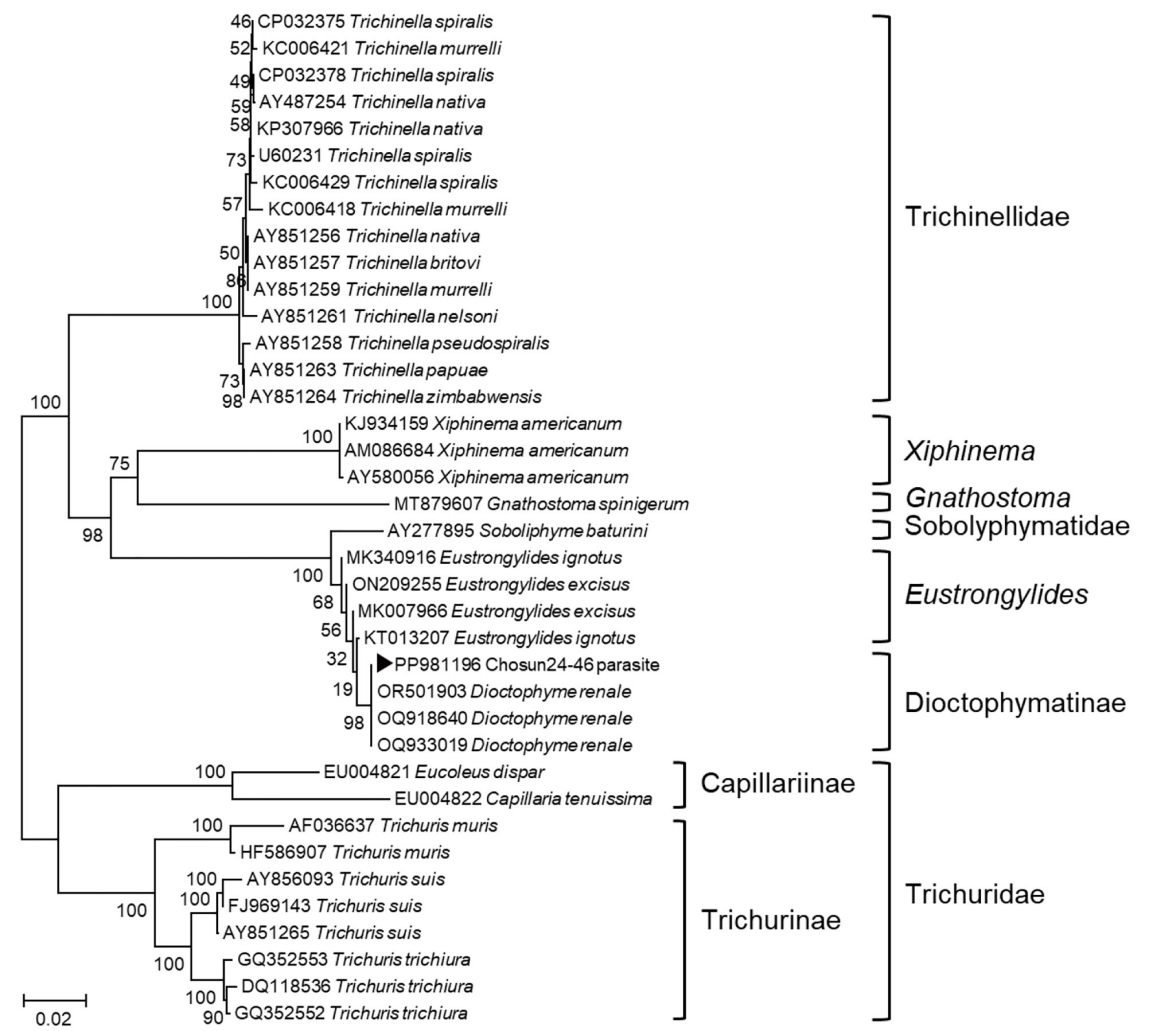
Phylogenetic tree for *Dioctophyme renale* roundworm showing a sequence from a patient with gastric submucosal tumor (black triangle), South Korea, 2024. Phylogenetic tree was based on the targeted 974-bp PCR amplicon sequence of *D. renale* from small subunit rRNA gene (18s RNA gene) sequences retrieved from GenBank (accession numbers shown). CLUSTAL X (http://www.clustal.org/clustal2) was used to construct phylogenetic tree by using neighbor-joining with 1,000 bootstrap replicates. We compared the *D. renale* sequence from our case with other roundworm sequences, and it aligned with 100% homology to *D. renale*. Scale bar indicates number of nucleotide substitutions per site.

To confirm the diagnosis, we performed cyclooxygenase 1 gene-specific PCR, which yielded negative results for both *D. renale* and *G. spinigerum*. However, nested PCR targeting the dorylipophorin gene showed 82.59% (503/609 bp) homology with *D. renale* (GenBank accession no. MW014827.1). Phylogenetic analysis of the small subunit rRNA gene clustered the sample with *D. renale*, supporting the final diagnosis of *D. renale* infection (Figure 3). For the dorylipophorin gene, only 1 registered sequence was available in GenBank; therefore, constructing a phylogenetic tree was not possible.

Patent infections by *D. renale* roundworms in humans are rare; the infections more often manifest as larval migrans with L3 larvae (*5*). The larvae mature into adults ≈6 months after ingestion. Although humans are definitive hosts for kidney infections, the larvae often erratically migrate. The larvae are found in the liver, abdominal cavity, and retroperitoneal space and as subcutaneous nodules (*6*). One study reviewed 37 global human *D. renale* cases and found 32 in the kidneys and 5 within the thigh, abdominal wall, and chest wall (*7*). The main clinical manifestations of human dioctophymiasis were loin pain (59.5%) and hematuria (59.5%) (*8*).

The long subcuticular polymyarian musculature, large boxy cells with prominent nuclei, and dark brown granules in the intestine distinguish *D. renale* from *Gnathostoma* spp. and *Anisakis* spp. roundworms (*8*). Ectopic migration of *D. renale* L3 larvae might lead to tumor-like masses in other organs, particularly in humans. In cases where roundworms manifest as gastric tumors, morphologic analysis and genetic confirmation are essential for accurate diagnosis.

## Conclusions

We confirmed *D. renale* giant kidney worm infection in this patient through both molecular and morphological analyses. The patient likely contracted the infection by eating freshwater fish, specifically the far-eastern catfish (*S. asotus*) and smelt (*H. olidus*). Conversely, *Anisakis* spp. infection typically occurs in raw saltwater fish. A study in Brazil identified a 53.2% prevalence of *D. renale* L3 larvae in *Hoplosternum littorale*, a freshwater catfish (*9*). Therefore, *H. olidus* catfish may serve as an intermediate host of *D. renale* roundworms in Korea. Further research on intermediate hosts is necessary. Because *D. renale* roundworms are found worldwide, regionally investigating intermediate hosts is crucial.

A limitation of this study is that we initially considered the parasite to be *Anisakis* spp.; thus, we obtained microscopic images of the entire specimen and immediately cut it for PCR analysis, which left no specimens for systematic morphologic analyses. In addition, the 603-bp dorylipophorin gene product was only 82.59% homologous to the *D. renale* sequence in GenBank. Some PCRs, including those targeting the cyclooxygenase 1 and dorylipophorin genes, failed to produce amplicons, possibly because of low DNA quality or primer mismatch. Further studies are needed to improve PCR conditions and confirm suitable genetic targets for *D. renale*. These findings might raise awareness among clinicians that *D. renale* infection can mimic anisakiasis and that PCR for certain gene targets may fail, emphasizing the need for careful morphologic evaluation and consideration of rare parasites in differential diagnoses.

In conclusion, although parasitic infections in humans are rare, atypical clinical manifestations may lead to diagnostic confusion among clinicians. When encountering roundworms in the submucosa or extragastrointestinal lesions, especially in patients with a history of raw fish consumption, clinicians should consider genetic testing alongside morphologic diagnosis to rule out giant kidney worms, anisakiasis, or gnathostomiasis. Accurate diagnosis of rare parasitic infections through molecular methods can prevent misdiagnosis and guide appropriate treatment.

Appendix Additional information for gastric submucosal tumor in patient infected with *Dioctophyme renale* roundworm, South Korea, 2024.
