# Supplementary material for: Gastric Submucosal Tumor in Patient Infected with Dioctophyme renale Roundworm, South Korea, 2024
Source: Emerg Infect Dis. 2025 Sep;31(9):1811–4. doi: 10.3201/eid3109.241944 (PMC12407218; doi:10.3201/eid3109.241944)
Supplement: Appendix — Additional information for gastric submucosal tumor in patient infected with Dioctophyme renale roundworm, South Korea, 2024. [file 24-1944-Techapp-s1.pdf]

Article DOI: https://doi.org/10.3201/eid3108.241944

EID cannot ensure accessibility for Supplemental Materials supplied by authors. Readers who have difficulty accessing supplementary content should contact the authors for assistance.

Gastric Submucosal Tumor in Patient Infected with *Dioctophyme renale* Roundworm, South Korea, 2024

Appendix

Appendix Table. List of primers and *Dioctophyme renale*– and *Gnathostoma spinigerum*–specific PCR results*

Target gene	Target species	Primers	Sequence 5′-3′	Size, bp	PCR	Reference	PCR result	GenBank accession no.
SSU rRNA	*Dioctophyme renale*	710F	ACTGCATCTTGGGCTTCGATG	1,150	N-PCR: 1st	This study		
		1860R	ACTTTCCCAACGCAACAGCC			This study		
		850F	ATGGAGAAGGACCTCGGTTC	890	N-PCR: 2nd	This study	Positive	PP981196
		1740R	ATCAGTGCGAGCTCATGACC			This study		
	Dioctophyme renale, Gnathostoma spinigerum	600F	AGAGGTGAAATTCTTGGACCG	690	N-PCR: 1st	This study		
		1290R	CAAAGGGCAGGGACGTAATC			This study		
		695F	GATCAGATACCGCCCTAGTTC	550	N-PCR: 2nd	This study	Positive	PV168478
		1245R	CTTACTGGGAATTCCTCGTTCAA			This study		
COX1	*Dioctophyme renale*	CO1_JB3-F	TTTTTTGGGCATCCTGAGGTTTAT	441	C-PCR	(*1*)	Negative	
		CO1_JB4.5-R	TAAAGAAAGAACATAATGAAAATG		N-PCR: 1st			
		DRCOX76F	TGGTGTGCTTGGTTGTTTTG	156	N-PCR: 2nd	(*2*)	Negative	
		DRCOX214R	AACCTGCCCACCATACAAAG					
		D.renale_COX1–10F	CATCCTGAGGTTTATATTCTAGC	410	N-PCR: 1st	This study		
		D.renale_COX1–420R	ATGACCCACTACATAATAAGTGTC			This study		
		D.renale_COX1–45F	GGTGTGATTTCTGAGAGAGTAAC	325	N-PCR: 2nd	This study	Negative	
		D.renale_COX1–370R	ATTCGCCAATGAAATGCCCGTTA			This study		
	Gnathostoma spinigerum	COX1_JB3–626F	TTTTTTGGTCATCCTGAAGTTTAT	441	N-PCR: 1st	(*1*)†		
		COX1_JB4.5–1065R	TAAACTTAAAACATAATGAAAATG					
		Gn_COI_F	GCCTGCTTTTGGAATTGTTAG	250	C-PCR	(*3*)	Negative	
		Gn_COI_R	ACGAAAACCATACAAAGTAGCCAA		N-PCR: 2nd		Negative	
Dorylipophorin	*Dioctophyme renale*	D.renale_dory-795F	GCTGTCACTGGCAAGTGTATA	1,249	N-PCR: 1st	This study		
		D.renale_dory-2044R	ACTTGCAATATTCCTGCGGTAC			This study		
		D.renale_dory-1037F	ACGCAGTGCTACAAGATGAGAG	626	N-PCR: 2nd	This study	Positive	Not submitted
		D.renale_dory-1663R	AGGTACTTCAAGATGTCGGCG			This study		
ITS2	*Gnathostoma spinigerum*	GS_ITS2_F	TGTGTCGATGAAGAACGCAG	650	C-PCR	(*3*)	Negative	
		GS_ITS2_R	TTCTATGCTTAAATTCAGGGG					

*C-PCR, conventional PCR; N-PCR, nested PCR; SSU, small subunit.
†Sequence modified to match the *G. spinigerum* sequence.

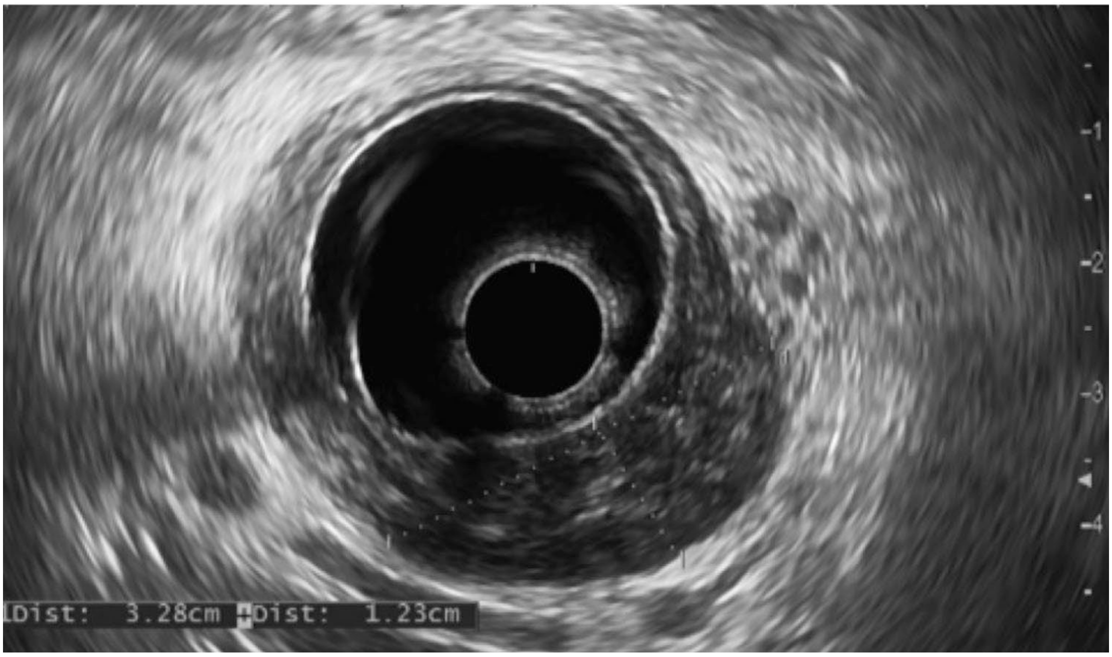

Appendix Figure 1. Endoscopic ultrasonography visualized diffuse gastric wall thickening and disruption of the submucosal layer in the proximal antrum of the gastric wall.

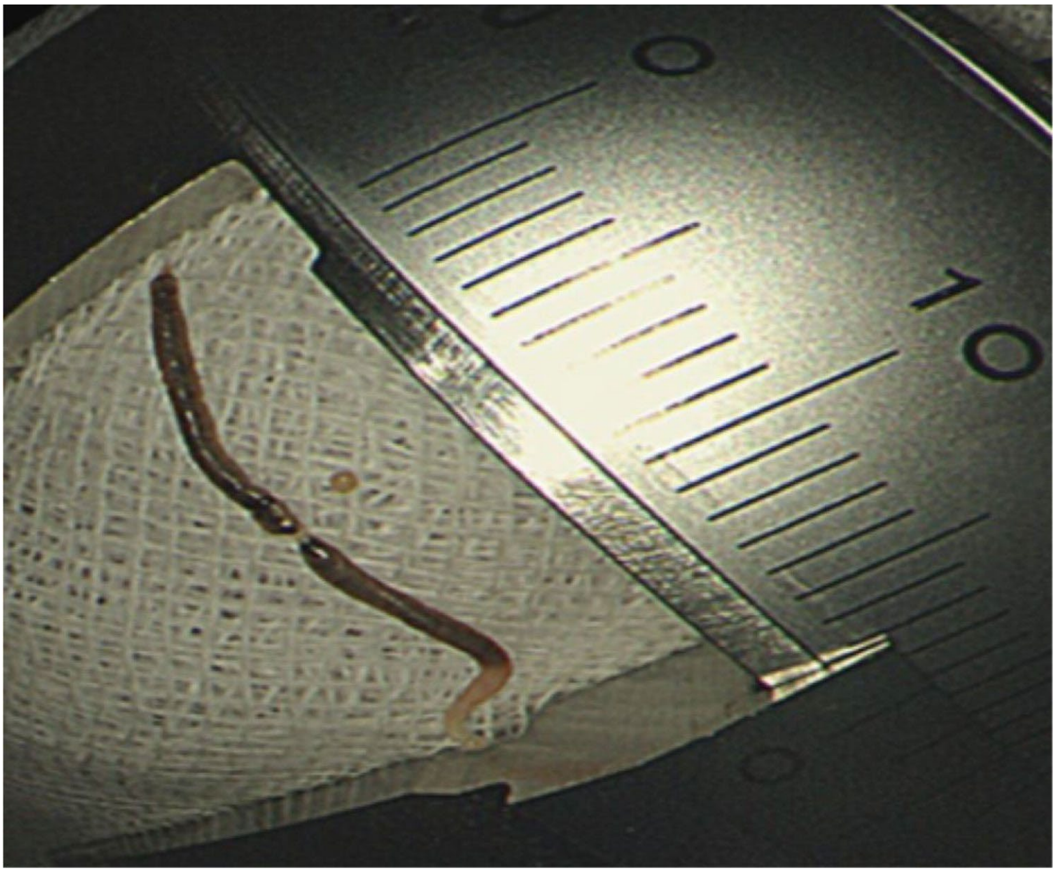

Appendix Figure 2. A dark red roundworm measuring ≈1.8 cm was removed with forceps during a biopsy.

References

1. Eslahi AV, Mowlavi G, Houshmand E, Pirestani M, Majidiani H, Nahavandi KH, et al. Occurrence of *Dioctophyme renale* (Goeze, 1782) in road-killed canids of Iran and its public health implication. Vet Parasitol Reg Stud Reports. 2021;24:100568. PubMed https://doi.org/10.1016/j.vprsr.2021.100568

2. Tokiwa T, Ueda W, Takatsuka S, Okawa K, Onodera M, Ohta N, et al. The first genetically confirmed case of *Dioctophyme renale* (Nematoda: Dioctophymatida) in a patient with a subcutaneous nodule. Parasitol Int. 2014;63:143–7. PubMed https://doi.org/10.1016/j.parint.2013.09.015

3. Jonthawin J, Intapan PM, Sanpool O, Sadaow L, Janwan P, Thanchomnang T, et al. Three human gnathostomiasis cases in thailand with molecular identification of causative parasite species. Am J Trop Med Hyg. 2015;93:615–8. PubMed https://doi.org/10.4269/ajtmh.15-0284
